# Surface sediment microbial communities remain viable, culturable, and metabolically active during sequential heating

**DOI:** 10.3389/fmicb.2026.1840877

**Published:** 2026-06-10

**Authors:** Falko Mathes, Erwan G. Roussel, Barry A. Cragg, Andrew J. Weightman, Henrik Sass, R. John Parkes, Gordon Webster

**Affiliations:** 1 School of Earth and Environmental Sciences, Cardiff University, Cardiff, Wales, United Kingdom; 2 UWA School of Agriculture and Environment, The University of Western Australia, Perth, WA, Australia; 3 Univ Brest, Ifremer, Biologie et Ecologie des Ecosystèmes marins Profonds (BEEP), Plouzané, France; 4 Microbiomes, Microbes and Informatics (MMI) Group, School of Biosciences, Cardiff University, Cardiff, Wales, United Kingdom

**Keywords:** culturability, deep biosphere, heat stress, metabolic activity, microbiome, necromass, viability

## Abstract

Marine sediments harbour a vast and diverse microbial biomass, yet it remains unclear how temperate surface sedimentary microbial communities transition to deep hot subsurface conditions. To simulate the effects of increasing temperature stress associated with deep burial, an estuarine surface sediment was sequentially heated from 15 °C to 90 °C over 434 days. Total cell counts increased during the first 210 days (42 °C), while FISH-detectable cells remained relatively constant for the first 56 days (15 °C). Overall culturability increased by one order of magnitude for heterotrophs and sulfate reducers, and by three orders of magnitude for methanogens. Subsequent heating from 42 °C to 90 °C resulted in a progressive decline in cell numbers, viability and culturability; however, culturable cells were detected throughout the experiment, including at the highest temperature (90 °C). Surprisingly, cells sampled at 90 °C were culturable across all incubation temperatures (15–90 °C) suggesting different members of the community were capable to grow across this large temperature range. Radiolabelled substrates were rapidly metabolised at 90 °C (≥1 day). Microbial community analysis demonstrated that with increasing temperature, members of the bacterial class Clostridia, mainly the Caldicoprobacteraceae (22.1%) and Peptococcaceae (10.5%) dominated the community. This study demonstrates that a phylogenetically diverse microbial community, originally adapted to temperate near-surface physicochemical conditions, can undergo functional and compositional restructuring and that certain members can become metabolically active under deep, thermally elevated sedimentary environments. This transition is likely mediated by the activation and selective enrichment of a cryptic thermophilic ‘seed bank’ present within the community and therefore may represent a mechanism of how deep sediments are inoculated with microbes and how the deep hot biosphere is sustained.

## 1 Introduction

Marine sediments are inhabited by vast numbers of microorganisms (Kallmeyer et al., 2012; Parkes et al., 2014; Bar-On et al., 2018) and living cells have been detected in deep and ancient sediments (Schippers et al., 2005; Inagaki et al., 2015; Morono et al., 2020; Beulig et al., 2022), where temperatures can reach 60 °C to 120 °C (Roussel et al., 2008; Ciobanu et al., 2014; Inagaki et al., 2015; Heuer et al., 2020; Beulig et al., 2022). The constraints exerted on cellular processes by these high temperatures and severe energy limitations in deep marine sediments (Jørgensen and D'Hondt, 2006; Lever et al., 2015) result in an overall decrease in biomass with increasing depth and geological time (Parkes et al., 1994; Parkes et al., 2014; Morono, 2023). Although the average thermal gradient in sediments is ~30 °C km^−1^, high temperatures are not exclusive to deep layers (Teske et al., 2014). Elevated temperature can affect microbial communities even in relatively shallow sediments (Mara et al., 2023), for example, where hydrothermal fluids emanate from the oceanic crust (Engelen et al., 2008), near hydrothermal vents (Tor et al., 2003; Møller et al., 2018), or in subductions zones (Beulig et al., 2022).

Increased temperatures conversely also help sustain the deep biosphere by providing substrates to the microbial communities (Teske et al., 2014). Lomstein et al. (2012) suggested that photosynthetically derived carbon can support *in situ* microorganisms for millions of years despite organic matter becoming more recalcitrant with depth (Jørgensen, 1982). This recalcitrant organic matter, however, can be activated at higher temperatures and low molecular weight compounds, such as acetate and hydrogen are produced (Wellsbury et al., 1997; Parkes et al., 2007) stimulating a number of metabolic processes such as methanogenesis, as well as metal and sulfate reduction (Roussel et al., 2015). Necromass produced as a result of temperature stress and starvation is preferentially degraded compared to the bulk organic matter (Lomstein et al., 2012; Braun et al., 2017; Bradley et al., 2018) and thus sustains surviving cells able to withstand the increasingly harsh conditions (Marshall et al., 2017). Furthermore, uncultured deep subsurface phyla employing biochemical mechanisms that increase cellular life spans, rely on syntrophic interactions and occupy different niches to survive (Bird et al., 2019).

It has been suggested that different ‘windows of opportunities’ exist at different temperatures for different sedimentary microbial processes (Roussel et al., 2015). To take advantage of these ‘windows’, microbial communities adapt their metabolic strategies to the environmental conditions. For example, it was shown that spores of thermophilic sulfate reducers deposited in cold, arctic sediments only germinate and become metabolically active at temperatures between 41 °C and 62 °C (Isaksen et al., 1994; Hubert et al., 2010). In contrast to this, Fichtel et al. (2012) isolated non-spore forming sulfate reducers from fluid-impacted sediments (~62 °C) of the Juan de Fuca Ridge. The wide temperature ranges for growth from 4 °C to 48 °C and metabolic versatility of the isolates suggested they survived as active populations throughout burial. In fact, many of the prominent prokaryotes inhabiting marine subsurface sediments, for example members of the phyla, Chloroflexota, Pseudomonadota, and Atribacterota (Parkes et al., 2014; Starnawski et al., 2017) and archaea (Takahata et al., 2001) cannot form spores and must endure unfavourable conditions in an inactive state while maintaining viability. How non-sporulating microorganisms achieve this is still not fully understood. Some cells may be metabolically inactive albeit repairing cellular damage to ensure survival (Price and Sowers, 2004) while others can be detected with methods assessing cellular viability (e.g., ribosomes by FISH; Biddle et al., 2006a, 2006b) or metabolic activity (Cragg et al., 1992; Beulig et al., 2022) suggesting that cells are ready to utilize substrates as soon as they become available. It has also been suggested that some members of the subsurface phylum, Atribacterota may survive by utilizing trehalose for biomolecular stabilization and employ an energy-efficient Na^+^-motive force to depress intracellular amino acid concentrations (Bird et al., 2019). Furthermore, the frequent isolation of Gram-negative bacteria (Süß et al., 2004; Biddle et al., 2005; Batzke et al., 2007; Süß et al., 2008; Fichtel et al., 2012; Wang et al., 2021) attests to their capability to survive in deeply buried nutrient-deprived sediments. Furthermore, it has been suggested that adaptive evolution is not a driving force in the deep biosphere but rather that survival of persistent taxa shapes the microbial community composition at depth (Starnawski et al., 2017; Dong et al., 2023).

Several studies have previously used a temperature-for-time displacement in sediment heating experiments to mimic processes in the laboratory that occur over thousands to millions of years in marine sediments (Wellsbury et al., 1997; Parkes et al., 2007; Parkes et al., 2011; Roussel et al., 2015). However, these experiments have mainly focussed on biogeochemical and microbial activity measurements, while information on how microbial viability and community composition changes with increasing temperature is lacking. The aim of this study was therefore to investigate how a near-surface estuarine sediment microbial community responds to increasing temperatures associated with burial in deep marine sediments over thousands to millions of years. We hypothesized that a near-surface community has the potential to change in response to increasing temperature stress, and that the changing environmental conditions may resuscitate dormant members of a microbial ‘seed bank’ (Lennon and Jones, 2011), enabling continued microbial viability and metabolic activity.

## 2 Materials and methods

### 2.1 Sediment collection, slurry preparation, and incubation

Sediment cores were collected from the Tamar estuary (Saint John’s Lake, 50°21′50.00″N, 4°14′10.68″W) in June 2009 using round, Perspex push cores (100 cm × 6 cm). Retrieved cores were cooled below *in situ* temperature (12 °C) and transported back to the laboratory in an upright position on ice. Two replicate 25% (v/v) large volume sediment slurries (2 L) were prepared in artificial seawater (Parkes et al., 2007) using the top 12 cm from two sediment cores. These replicates were designated as Slurry I (SI) and Slurry II (SII) throughout this study. Slurries were incubated under anoxic conditions in the dark in modified gas-tight, conical flasks fitted with gas (top) and liquid (bottom) sampling ports (Parkes et al., 2007). For the first 56 days, the slurry was incubated at 15 °C after which the temperature was increased by 3 °C every 14 days until 90 °C was reached. After 14 days at this final temperature, the total incubation time was 434 days and spanned the temperature ranges for mesophilic (10 °C–40 °C), thermophilic (40 °C–70 °C) and hyperthermophilic organisms (>70 °C).

### 2.2 Biogeochemical analyses

Headspace gas and pore water were sampled every 2 weeks. Carbon dioxide, methane, and hydrogen concentrations were determined using a natural gas analyzer (PerkinElmer Clarus 500) while volatile fatty acid (VFA) and sulfate concentrations were quantified by ion chromatography (Dionex™ ICS-2000) as described (Webster et al., 2010).

### 2.3 Direct cell counts

Acridine orange direct counts (AODC) were performed according to Fry (1988). Samples were fixed with filter-sterilised (0.2 μm) 2% (v/v) formaldehyde solution in 3.5% NaCl in particle free, glass vials and stored in the dark at room temperature (RT) until processing. For counting, a subsample (115 μL) of fixed slurry was diluted in 10 mL formaldehyde, stained with 50 μL acridine orange (1 g L^−1^) for three minutes, and filtered onto black polycarbonate membrane filters (0.2 μm). Filters were mounted onto glass slides using paraffin oil and viewed by epifluorescence microscopy (Axioskop I, Zeiss).

Samples for fluorescence *in situ* hybridisation (FISH) were fixed in phosphate buffered saline solution (PBS: 145 mM NaCl, 1.4 mM NaH_2_PO_4_, 8.0 mM Na_2_HPO_4_, pH 7.4) containing 4% (v/v) formaldehyde (Glöckner et al., 1996; Glöckner et al., 1999). Samples were then washed three times with PBS and stored in a 1:1 mixture of ethanol and PBS at −20 °C until further analysis. Hybridisation was performed according to Pernthaler et al. (2002) and Ishii et al. (2004). Cells were filtered onto white polycarbonate filters (Isopore Membrane filters, 0.2 μm GTTP; Merck Millipore), dehydrated using a sequence of ethanol baths (50% for 5 min, 80% for 1 min, 90% for 1 min) and dried at RT. Filters were covered with probe solution (EUB338-I or NON-EUB338, 50 ng μL^−1^; Glöckner et al., 1996) in 8 μL of hybridisation buffer (0.9 M NaCl, 20 mM Tris–HCl [pH 7.4], 35% (w/v) formamide, 0.01% (w/w) SDS), transferred onto glass slides and incubated at 46 °C for 2–4 h (Hybridisation Oven S101HS, Stuart Scientific). Filter sections were transferred into preheated washing buffer (70 mM NaCl, 20 mM Tris–HCl [pH 7.4], 5 mM EDTA, 0.01% (w/v) SDS) and incubated for 20 min at 48 °C. Filters were covered with 50 μL DAPI solution (1 μg mL^−1^) for 10 min in the dark at RT and gently washed in 0.2 μm filtered ddH_2_O and dried. All filters were mounted onto glass slides using *SlowFade*^®^ Gold (Invitrogen Corporation) for microscopy.

AODC and FISH cell counts were performed in duplicate or triplicate, and at least 200 cells or 20 randomly selected fields of view were counted. Cells on and off sediment particles were recorded separately to adjust counts for cells hidden from view (Goulder, 1977).

### 2.4 Culturable cell counts

To determine the number of culturable anaerobic microorganisms, anoxic basal growth media were prepared according to Süß et al. (2004). To stimulate growth of different metabolic groups the following substrates were added: glucose (1 mM) for heterotrophs, Na-acetate and Na-lactate (10 mM each) for sulfate reducers, and Na-acetate (10 mM) without sulfate for methanogens. For each target group, three parallel dilution series were prepared in 96-deep-well plates (Beckman Coulter; Süß et al., 2004). Under anoxic conditions (Modular Atmosphere Controlled System; Don Whitley Scientific) 100 μL of slurry were added to 900 μL of growth medium, diluted (up to 10^−8^) and incubated in sealed plastic bags containing Anaerocult^®^ A mini (Merck) in the dark at different temperatures (15 °C to 90 °C). Note as Anaerocult^®^ A releases hydrogen (Heizmann and Werner, 1989) hydrogentrophic microorganisms (sulfate reducers, methanogens, acetogens) would also be stimulated. Growth was confirmed using the Sybr®Green I assay as described (Martens-Habbena and Sass, 2006). From each MPN well, 200 μL subsamples were transferred into black microtiter plates (Nunc™ F96 Microwell, Nunc GmbH & Co. KG) and mixed with 50 μL of Sybr^®^Green I solution (Invitrogen Corporation; diluted 1:2000 in TE buffer [200 mM Tris–HCl, 50 mM sodium EDTA, pH 8.0]). Plates were incubated in the dark and fluorescence measured after 4 and 20 h (Excitation 485 nm, Emission 530 nm) using a plate reader (Fluorocount BF10001, Packard BioScience). To detect growth in MPN wells, fluorescence measurements of uninoculated blanks were averaged, the standard deviation calculated and five times this value was added to the mean blank. This value was then subtracted from the sample wells, and if resulting value was greater than zero the well was scored positive for growth. Using the tables of de Man (1983) the most probable number of culturable cells for the original sample was determined.

### 2.5 DNA extraction

Genomic DNA was extracted in triplicate from sediment slurry samples using the FastDNA® SPIN Kit for Soil (MP Biomedicals) as described (Webster et al., 2003). Briefly, 2 mL of sediment slurry were placed in a lysing matrix E tube (MP Biomedicals) and centrifuged at 15,000 × *g* for 5 min to pellet cells and sediment. Sediment pellets were then re-suspended in 800 μL of sodium phosphate buffer and 120 μL MT buffer (MP Biomedicals) before lysis in a FastPrep^®^ 24 instrument (MP Biomedicals) for 2 × 30 s at speed 5.5 ms^−1^. All remaining steps were conducted as per the manufacturer’s protocol, except that some spin and incubation times were extended (see Parkes et al., 2010 for a detailed description). DNA was eluted in 100 μL molecular grade water (Severn Biotech) and stored at −80 °C until processing.

### 2.6 Microbial community analysis

To assess the microbial community succession of the heated slurry, polymerase chain reaction (PCR) was conducted with slurry DNA samples using primers 515F and 806R (Caporaso et al., 2011) to amplify the V4 region of the 16S rRNA gene. PCR included a non-template (i.e., negative) control as well as a positive control. In addition to the “core primers,” the forward primer was modified as described (Whiteley et al., 2012) to contain a sequencing adaptor, a “GT” spacer, and a unique Golay barcode sequence (Hamady et al., 2008) to allow multiplexing of samples. The reverse primer was extended by the P1 adapter sequence that is used to attach the DNA to the ion sphere particle (Whiteley et al., 2012) of the Ion Torrent Personal Genome Machine (Life Technologies). The PCR master mix (Caporaso et al., 2011) consisted of 8.56 μL PCR Grade H_2_O, 8.0 μL 5 Prime Hot MM (VWR International), 1.0 μL barcoded forward primer (4 μM), 1.0 μL primer mix consisting of the P1 reverse primer (4 μM), and the core forward and reverse primer (0.44 μM), 0.24 μL BSA (50 μg μL^−1^), and 1.0 μL template DNA (1 ng μL^−1^). PCR was conducted in a thermocylcer (Eppendorf) under the following conditions: initial denaturing 94 °C for 2 min, 25 cycles of denaturing at 94 °C for 45 s, annealing at 50 °C for 60 s, elongation at 65 °C for 90 s. This was followed by 5 cycles of denaturing at 94 °C for 45 s and annealing/elongation at 65 °C for 90 s. Finally, a final elongation step at 72 °C for 10 min was conducted prior to cooling to 10 °C.

Successful PCR amplification was confirmed by agarose gel (1.5%) electrophoresis stained with ethidium bromide. DNA concentrations were quantified using the Qubit^®^ 2.0 Fluorometer (Life Technologies). Individual samples were pooled in equimolar ratios purified by gel electrophoresis (80 V for 50 min). The DNA band was cut and purified using the Wizard^®^ SV Gel and PCR Clean-Up System (Promega) following the manufacturers protocol. Next generation sequencing was conducted at the Lotterywest State Biomedical Facility Genomics (LSBFG, UWA) using an Ion Torrent Personal Genome Machine (Life Technologies Australia Pty Ltd.; Rothberg et al., 2011) as described (Whiteley et al., 2012) using the 400 base pair chemistry. Obtained sequences were analysed using QIIME 1.8.0 (Caporaso et al., 2010). Multiplexed, barcoded sequences were split and only sequences with a minimum quality score of 20 containing no mismatches in the barcode, forward, and reverse primer were retained. Chimeras were removed using Usearch (Edgar, 2010). Subsequently, all sequences were clustered into operational taxonomic units (OTUs) based on 97% sequence similarity and taxonomy was assigned using the SILVA database (Quast et al., 2013). Singletons were removed and sequences were then rarefied to minimum 56,300 sequences per sample to allow for comparison between samples with different sequencing depth (Gihring et al., 2012). Sequencing data has been deposited under QIITA study ID 11674 (qiita.ucsd.edu; Gonzalez et al., 2018) and the European Nucleotide Archive study accession PRJEB30259 ^1^.

### 2.7 Quantitative PCR of Bacteria and Archaea 16S rRNA genes

Quantitative PCR (qPCR) was used to quantify 16S rRNA gene copy numbers of Bacteria and Archaea in slurry samples. SybrGreen chemistry was used for all protocols (Webster et al., 2015). All qPCR reactions for standards, non-template controls, and sediment slurry samples were undertaken in triplicate and amplified on an Agilent Mx3000P QPCR System (Agilent Technologies UK Ltd.). For standard curves and calibration, serial dilutions of full length 16S rRNA gene PCR products from *Anaerolinea thermophila* DSM 14523 and *Methanococcoides methylutens* DSM 2657 were used as standards for Bacteria and Archaea, respectively. All standard PCR products were cleaned using Merck Millipore Microcon™ Centrifugal Filters and quantified with a Qubit Fluorometer (Invitrogen), and numbers of 16S rRNA gene copies calculated as described (Smith et al., 2006). To ensure good quantification data, qPCR results were rejected if the r^2^ value of the standard curve was below 0.95 or the efficiency of the reaction was below 90%. The qPCR mixtures for all reactions (standards, controls and samples) were contained in a total volume of 20 μL with 400 nM of each primer (Eurofins MWG) and 1 μL of DNA in 1x qPCRBIO SyGreen Lo-ROX Mix (PCR Biosystems Ltd.) made up with molecular grade water (Severn Biotech Ltd.). 16S rRNA gene primers 534F/907R (GCCAGCAGCCGCGGTAAT/CCGTCAATTCCTTTGAGTTT) and S-D-Arch-0025-a-S-17F/S-D-Arch-0344-a-S-20R (CTGGTTGATCCTGCCAG/TCGCGCCTGCTGCGCCCCGT) were used to target Bacteria and Archaea, respectively (Webster et al., 2015). The qPCR protocol was 95 °C for 7 min, 40 cycles of 95 °C for 30 s, 52 °C for 30 s, 72 °C for 60 s, followed by a melting curve from 55 °C to 95 °C. Each cycle was followed by data acquisition at the elongation step.

### 2.8 Potential metabolic activity

Potential metabolic activity after heating the sediment slurry up to 90 °C was determined using D-[U-^14^C] glucose, DL-[U-^14^C] lactate, and [U-^14^C] acetate (total activity of 1.85 MBq, American Radiolabeled Chemicals). For this, three millilitres of slurry were transferred into 10 mL gas-tight autoclaved serum vials, and flushed with N_2_/CO_2_ (80/20, v/v). A batch of the radioactive stock solution was diluted (1:10) and 50 μL were added to slurries resulting in final concentrations of 2.2 nM for glucose (72.5 kBq), 3.8 nM lactate (78.7 kBq), and 6.7 nM acetate (23.4 kBq). Slurries were incubated in the dark at 90 °C for up to 30 days. This incubation time was chosen as MPN plates showed positive growth in the multi-well plates after up to 4 weeks. For analysis, a subsample of 1.5 mL was taken and 0.5 mL NaOH (2.0 M) was added to the remaining 1.5 mL of culture to stop activity and to bring any gaseous CO_2_ into solution for later analysis. From the subsample, 0.1 mL were transferred into a glass scintillation vial (Lablogic Systems) and 9.9 mL InstaGel Plus (Perkin Elmer) were added. Another 0.1 mL were diluted (1:10) and centrifuged for 6 min at 15,000 × *g* to separate pore water and sediment particles. The supernatant was transferred into a vial for chromatographic separation and fraction collection using a Dionex™ ICS-2000 Ion Chromatography System equipped with a Foxy Jr.^®^ fraction collector (Teledyne Isco) followed by liquid scintillation counting as described (Roussel et al., 2015). Different fractions were collected in polyethylene vials (Perkin Elmer) containing 0.5 mL NaOH (1 M), transferred into glass scintillation vials and 9.9 mL InstaGel were added. To determine the amount of carbon incorporated into microbial biomass, three parallels of 0.3 mL slurry were filtered onto Cyclopore™ track etched, polycarbonate, hydrophilic filter membranes (0.2 μm, Whatman) and washed with 10 mL PBS (pH 7.3) to remove any radioactivity present outside the cells. Filter membranes were then transferred into plastic scintillation vials (Perkin Elmer) containing 10 mL liquid scintillation fluid (Scintisafe 3, Fisher Scientific). To quantify CO_2_ production, 1 mL of the remaining slurry to which NaOH was added was transferred into gastight serum vials containing 4 mL HCl (1 M) and a magnetic flee. The headspace was flushed with nitrogen whilst stirring to remove all CO_2_ from the vial. The gas phase was passed through ultrapure H_2_O (>18 MΩ·cm) amended with VFAs and acidified (pH > 3) before it was passed through three consecutive batches of scintillation fluid (10 mL) containing 7% phenethylamine (Sigma-Aldrich) to capture the gaseous CO_2_. All vials containing InstaGel or scintillation fluid were measured using a liquid scintillation counter (TRI-CARB 2900TR, Perkin Elmer). Determination of DPM in vials with InstaGel (fractions and plain sample) was conducted three times for 20 min, whereas the filter membranes and trapped CO_2_ in liquid scintillation fluid were measured twice for 10 min. For each radioactively labelled substrate, one autoclaved (121 °C for 15 min) parallel incubation was designed to serve as a putative abiotic control. These controls were incubated for 30 days and then processed as described above.

### 2.9 Statistical analysis

Similarity percentage (SIMPER), principal component analyses, and Permutational Multivariate Analysis of Variance (PERMANOVA) were conducted in PRIMER-E (Clarke, 1993) using the taxonomy assignments at the genus level. For cell counts and qPCR gene copy numbers, Pearson’s correlation and Spearman’s rank coefficient analyses with Benjamini-Hochberg false discovery detection as well as repeated measures ANOVA with Bonferroni-corrected post-hoc tests were conducted in Microsoft Excel using the Real Statistics Resource Pack.^2^

## 3 Results

### 3.1 Changes in slurry biogeochemistry during sequential heating

**Figure 1:**
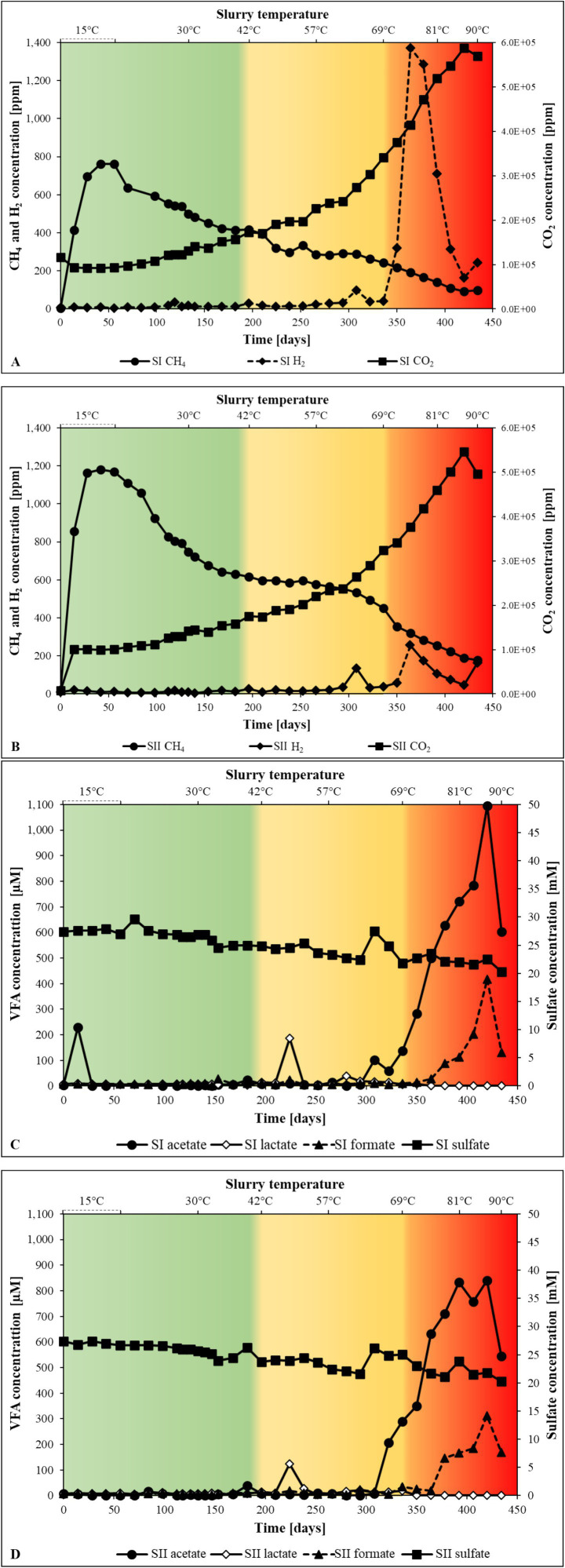
Biogeochemistry of sediment slurries during sequential heating. **(A,B)** Headspace gas concentrations of the two parallel incubated slurries I and II, respectively. (**C**,**D**) Volatile fatty acids and sulfate concentrations for slurries I and II, respectively. Slurry I = SI, Slurry II = SII. Colored background indicates meso- (green), thermo- (yellow), and hyperthermophilic (red) temperature ranges. For the first 56 days, the slurry was maintained at 15 °C as denoted by the black dashed line, and then the temperature was increased by 3 °C every 14 days until 90 °C.

During the first 56 days of incubation at 15 °C, methane concentrations increased in the headspace of the two slurries from 3 ppm to 762 ppm for slurry I (SI) and from 22 ppm to 1,179 ppm for slurry II (SII; Figures 1A,B). After this initial period of 56 days, sequential heating commenced and methane concentrations decreased by 9 ppm per day until 70 days in slurry I (18 °C) and by 6.2 ppm per day until 112 days in slurry II (27 °C), respectively. After this, the decrease continued at a lower rate until the final temperature of 90 °C was reached after 434 days (Figures 1A,B). While in slurry I this decrease was consistently around 1.4 ppm per day, in slurry II two phases were identifiable with the first phase (day 112 to 294) showing hardly any methane decrease. After day 294 (equivalent to 60 °C) methane consistently decreased at a rate of 2.9 ppm per day. Concomitantly, carbon dioxide concentrations increased in the headspace (Figures 1A,B) likely caused by microbial activity but intensified by the increased slurry temperature decreasing CO_2_ solubility (Roussel et al., 2015). Interestingly, the rate of CO_2_ production increased with temperature. Concentrations of H_2_ remained relatively low until 294 days (60 °C) before a maximum occurred in both slurries after 308 days (63 °C) and at day 364 (75 °C), respectively (Figures 1A,B). Although H_2_ concentrations varied between the two slurries, these changes occurred at the same time points.

Volatile fatty acids and sulfate profiles were similar between the two slurries (Figures 1C,D) but showed more variation than the gas concentrations. Acetate concentrations showed a local maximum after 2 weeks (15 °C) of incubation in slurry I (230 μM) but remained low (5.9 μM) in slurry II. Between 28 (15 °C) and 294 days (60 °C), acetate concentrations remained relatively constant averaging 4.1 μM in slurry I and 5.0 μM in slurry II, respectively. This was followed by a steady increase to 1,096 μM in slurry I and 840 μM in slurry II after 420 days (87 °C) before decreasing to 603 μM in slurry I and 546 μM in slurry II over the final 2 weeks. Lactate concentrations were on average 8.2 μM and did not increase with increasing temperature except after 224 days (48 °C) when maxima occurred in both slurries (187 μM in slurry I and 123 μM in slurry II; Figures 1C,D). Formate remained low for most of the experiment with an average concentration of 10 μM between 0 (15 °C) and 364 days (75 °C). Subsequently, concentrations increased to 417 μM in slurry I and 312 μM in slurry II after 420 days (87 °C) before decreasing again to 131 μM in slurry I and 170 μM in slurry II during the final 2 weeks of the experiment (Figures 1C,D).

Overall, sulfate concentrations decreased over time and with increasing temperature from 27 mM at day 0 (15 °C) to 20 mM after 434 days in both slurries (90 °C, Figures 1C,D). However, there were a few noteworthy increases in sulfate concentrations in the meso-, thermos-, and hyperthermophilic temperature ranges (Figures 1C,D). Notwithstanding the local sulfate maxima, sulfate was reduced at a rate of 0.022 mmol L^−1^ d^−1^ in both slurries between days 98 and day 294 spanning both the mesophilic and thermophilic temperature range (24 °C to 60 °C). During the experiment, there was an average total loss of 19.4 mM of sulfate while a total average of 12.3 mM was produced in both slurries. This resulted in an average net loss of 7.1 mM of sulfate. Since the biogeochemical profiles of the two parallel-incubated replicate slurries were very similar, we focused our investigation of the microbial community response to sequential heating on slurry I.

### 3.2 Cell counts, viability, and culturability during sequential heating

**Figure 2:**
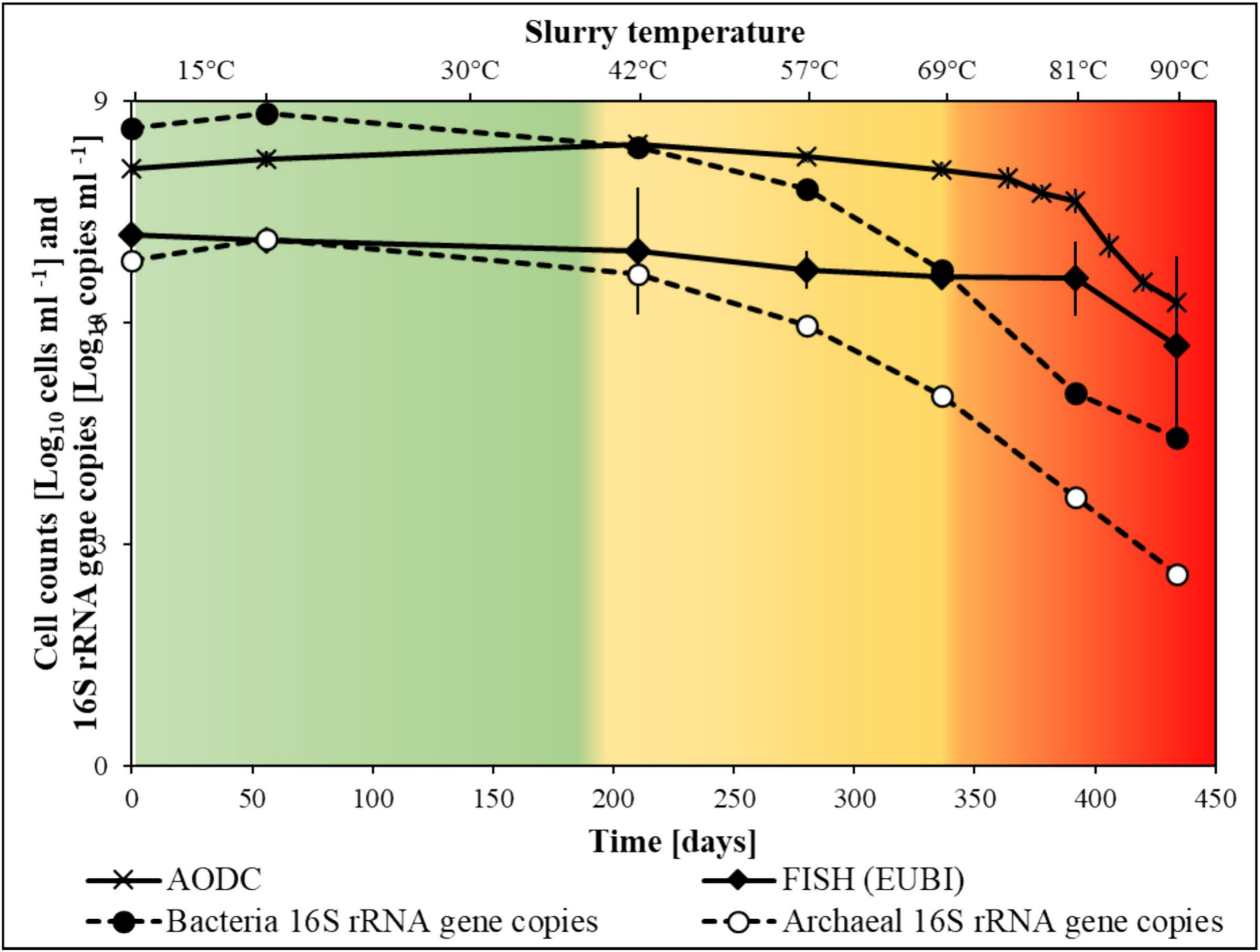
Cell counts and 16S rRNA gene abundances during sequential heating. Error bars represent 95% confidence limits; if not visible, they fall within the data point. Colored background indicates meso- (green), thermo- (yellow), and hyperthermophilic (red) temperature ranges. EUBI, EUB338-I probe.

Total cell counts (AODC) increased by 35% (*p* = 0.065) between 0 and 56 days (15 °C, from 1.2 × 10^8^ to 1.6 × 10^8^ cells mL^−1^; Figure 2, Table 1) presumably due to the mixing of previously stratified sediments and the resulting homogenous distribution of substrates, nutrients, and electron acceptors throughout the sediment slurry. During sequential heating across the mesophilic temperature range, a further increase to 2.5 × 10^8^ cells mL^−1^ occurred (between day 56 and day 210 [42 °C], *p* = 0.002). Subsequently, cell numbers decreased culminating in a total cell loss of 99.3% or two orders of magnitude between day 210 (42 °C, 2.5 × 10^8^ cells mL^−1^) and day 434 (90 °C, 1.9 × 10^6^ cells mL^−1^, *p* < 0.001). Cell counts were significantly negatively correlated with increased temperature (Spearman’s rho = −0.925, *p* < 0.0001). However, the decline can be divided into three stages (Table 1). During the first phase of decline (day 210 and day 364 [75 °C]), a net decrease in cell counts by 5.0 × 10^6^ cells mL^−1^ K^−1^ occurred (from 2.5 × 10^8^ to 8.9 × 10^7^ cells mL^-1,^ 65% decrease), showing a statistically significant negative correlation between increasing temperature and decreasing cell count (n = 4; *p* = 0.008; Table 1). Subsequently, the rate of net cell loss increased to 7.5 × 10^6^ cells mL^−1^ K^−1^ during the second phase of decline between 364 days (75 °C) and 392 days (81 °C) resulting in a residual population of 4.4 × 10^7^ cells mL^−1^ (51% decrease). During this phase, there was a strong correlation between increasing temperature and decreasing cell counts, however, this was not significant due to the low number of samples in this phase (*n* = 3; *p* > 0.05; Table 1). Net cell loss during the third phase between 392 and 434 days (90 °C) resulted in a final cell count of 1.9 × 10^6^ cells mL^−1^ (96% decrease) and was significantly correlated with temperature (*n* = 4; *p* = 0.013). However, the rate (4.5 × 10^6^ cells mL^−1^ K^−1^) was lower compared with the two previous phases of decline (Table 1), which can be explained by the low cell numbers at the start of this phase.

**Table 1:** Total cell counts and phases of decline.

Time [days]	Temperature [°C]	AODC [cells mL^−1^]	Percent change compared to t_0_	Phase of decline	Rate of decrease [cells mL^−1^ K^−1^], decrease [%], and r^2^
0	15	1.2 × 10^8^	**100.0%**	n.a.	n.a.
56	15	1.6 × 10^8^	135.3%	n.a.	n.a.
210	42	2.5 × 10^8^	212.0%	1	5.0 × 10^6^, −65.0% r^2^ = 0.992*
280	57	1.7 × 10^8^	145.3%	1	
336	69	1.2 × 10^8^	99.2%	1	
364	75	8.9 × 10^7^	74.2%	1 and 2	7.5 × 10^6^, −50.5% r^2^ = 0.991
378	78	5.8 × 10^7^	48.1%	2	
392	81	4.4 × 10^7^	36.7%	2 and 3	
406	84	1.1 × 10^7^	9.2%	3	4.5 × 10^6^, −95.8% r^2^ = 0.987*
420	87	3.5 × 10^6^	2.9%	3	
434	90	1.9 × 10^6^	1.6%	3	

Phase of decline 1: *n* = 4; phase 2: *n* = 3; phase 3: *n* = 4. n.a. = not applicable; * = statistically significant correlation after Benjamini–Hochberg tests for false discovery rate. Phases of decline are defined as significant decreases in cell numbers.

In contrast to total cells counts, FISH detectable cells only decreased between day 0 (15 °C, 1.5 × 10^7^cells mL^−1^) and day 434 (90 °C, 5.1 × 10^5^ cells mL^−1^; Spearman’s rho = −0.953; *P* = <0.001; Figure 2). The rate of linear decrease between day 56 (15 °C) and day 392 was estimated to 3.0 × 10^4^ cells mL^−1^ K^−1^ and significantly correlated to increasing temperature (*p* = 0.005). This was followed by a drastic decrease (87%) between day 392 and day 434 (90 °C, Figure 2) from 4.0 × 10^6^ cells mL^−1^ to 5.1 × 10^5^ cells mL^−1^, respectively, at a rate of 8.3 × 10^4^ cells mL^−1^ K^−1^.

Throughout the experiment, the abundance of bacterial 16S rRNA genes determined by qPCR was almost two orders of magnitude higher than for Archaea even at hyperthermophilic temperatures. Despite this, their temporal trends were similar (Figure 2) both increasing between day 0 (15 °C, 4.4 × 10^8^ and 6.8 × 10^6^ copies mL^−1^, respectively) and day 56 (15 °C, 6.9 × 10^8^ and 1.4 × 10^7^ copies mL^−1^, respectively) and then decreasing until the end of the experiment (90 °C, 2.7 × 10^4^ and 3.9 × 10^2^ copies mL^−1^, respectively). Overall 16S rRNA gene copy numbers for both Bacteria and Archaea were significantly correlated with increasing temperatures (Spearman’s rho = −0.953; *p* = 0.001). During the first stage of linear decline between 15 °C and 57 °C, decreases in copy numbers occurred at a rate of 1.5 × 10^7^ copies mL^−1^ K^−1^ for Bacteria and 3.0 × 10^5^ copies mL^−1^ K^−1^ for Archaea. The second stage between 57 °C and 90 °C showed lower rates (1.9 × 10^6^ copies mL^−1^ K^−1^ for Bacteria and 2.6 × 10^4^ copies mL^−1^ K^−1^ for Archaea) due to the lower abundance of copy numbers at the start of this phase.

**Figure 3:**
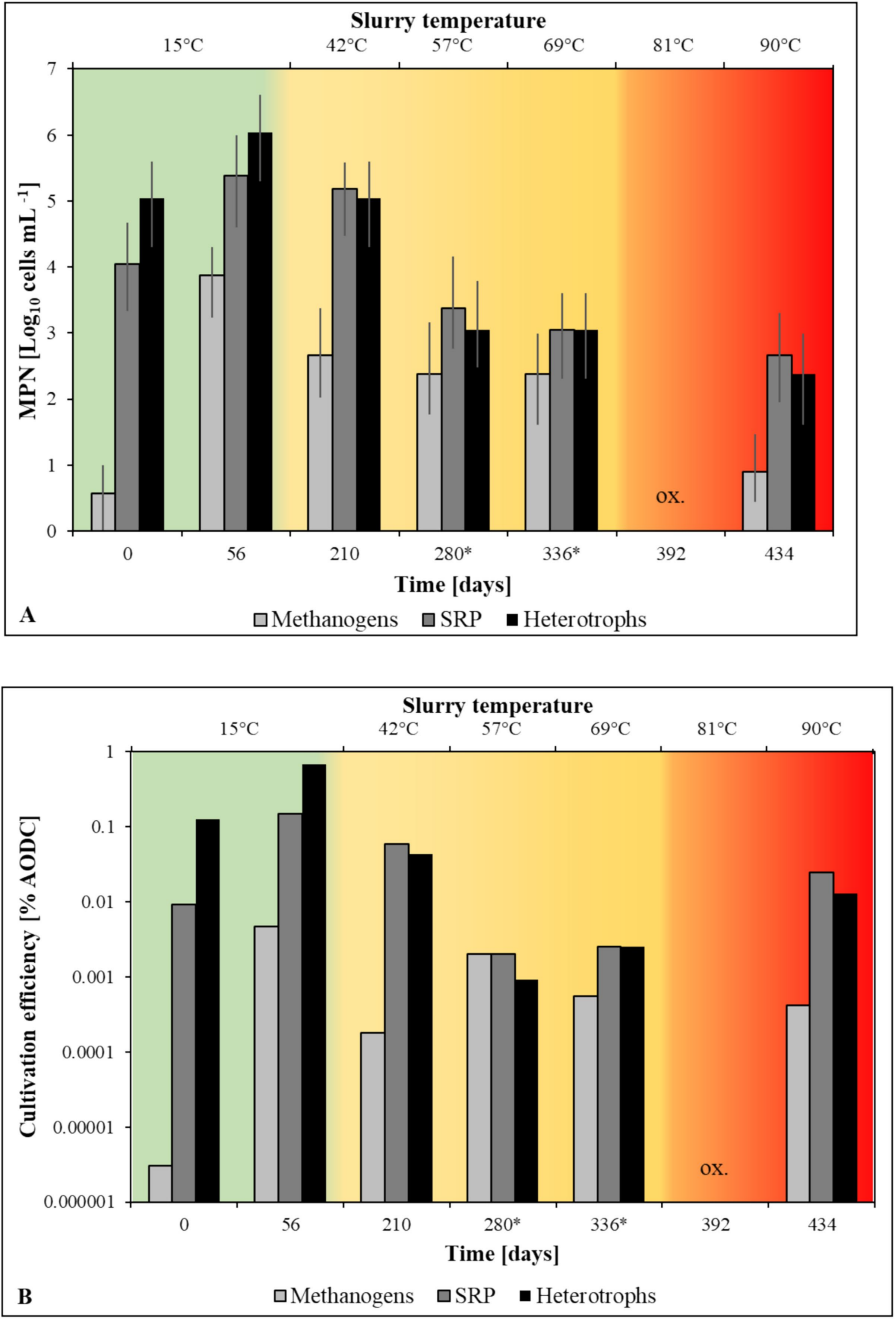
Prokaryotic culturability during sequential heating. **(A)** MPN counts of sediment slurry. **(B)** Cultivation efficiencies based on AODC direct counts. MPN plates were incubated at the temperature at the time of sampling the sediment slurry. Error bars represent 95% confidence limits. *Data for 210 days (42 °C) was obtained from subsequent timepoint (336 days, 69 °C) incubated at 42 °C, and data for 336 days (69 °C) was obtained from subsequent timepoint (81 °C) incubated at 69 °C due to the MPN multiwell plates oxidising during incubation. This oxidation problem also occurred with the MPN multiwell plate incubated at 81 °C hence no data is available (ox.). Colored background indicates meso- (green), thermo- (yellow), and hyperthermophilic (red) temperatures ranges.

Similar to the total counts and abundance of gene copy numbers, culturability of all three target metabolic groups increased between day 0 (15 °C) and day 56 (15 °C, Figure 3). While MPN counts for sulfate reducers and heterotrophs increased by one order of magnitude (from 1.1 × 10^4^ to 2.4 × 10^5^ cells mL^−1^ and from 1.5 × 10^5^ to 1.1 × 10^6^ cells mL^−1^, respectively), MPN counts targeting methanogens considerably increased by three orders of magnitude (from 4.0 cells mL^−1^ to 7.5 × 10^3^ cells mL^−1^, Figure 3A). Likewise, cultivation efficiencies also increased for all three groups during the first 56 days (Figure 3B, Supplementary Table 1). With increasing temperatures, MPN counts for all three metabolic groups decreased but remained above the detection limit (Figure 3A). Unfortunately, the MPN plate incubated at 81 °C became oxidised (Figure 3) due to the plastic bag containing the deep-well plate and the Anaerocult® sachet opening during incubation, likely as a result of temperature-induced gas expansion within the bag. Precautions were taken for this not to re-occur for the highest temperature tested. Final MPN counts after 434 days (90 °C) were 8.0 cells mL^−1^ with methanogen medium, 460 cells mL^−1^ for sulfate reducers, and 240 cells mL^−1^ for heterotrophs. Cultivation efficiencies based on total cell counts were generally low (Figure 3B). The highest value was obtained for sulfate reducers sampled at 90 °C slurry temperature and incubated at 40 °C with a cultivation efficiency of 13% (Supplementary Figure 1B). In contrast to the progressive decline in MPN counts from 56 days (15 °C) onwards, cultivation efficiencies for SRPs (sulfate-reducing prokaryotes) and heterotrophs were one order of magnitude higher after 434 days (90 °C) than after 336 days (69 °C) (Supplementary Figure 1B) indicating survival and, due to continuing sulfate consumption, activity of a hyperthermophilic subpopulation. Surprisingly, culturable cells were detected at all incubation temperatures, even after heating the sediment slurry to 90 °C, (Supplementary Figure 1). Sulfate reducers showed a maximum of 2.4 × 10^5^ cells mL^−1^ at 40 °C which was similar to MPNs after 210 days (42 °C) and 280 days (57 °C, Supplementary Figure 1) indicating they remained viable at hyperthermophilic temperatures, perhaps surviving by forming spores.

### 3.3 Changes in microbial community composition during sequential heating

**Figure 4:**
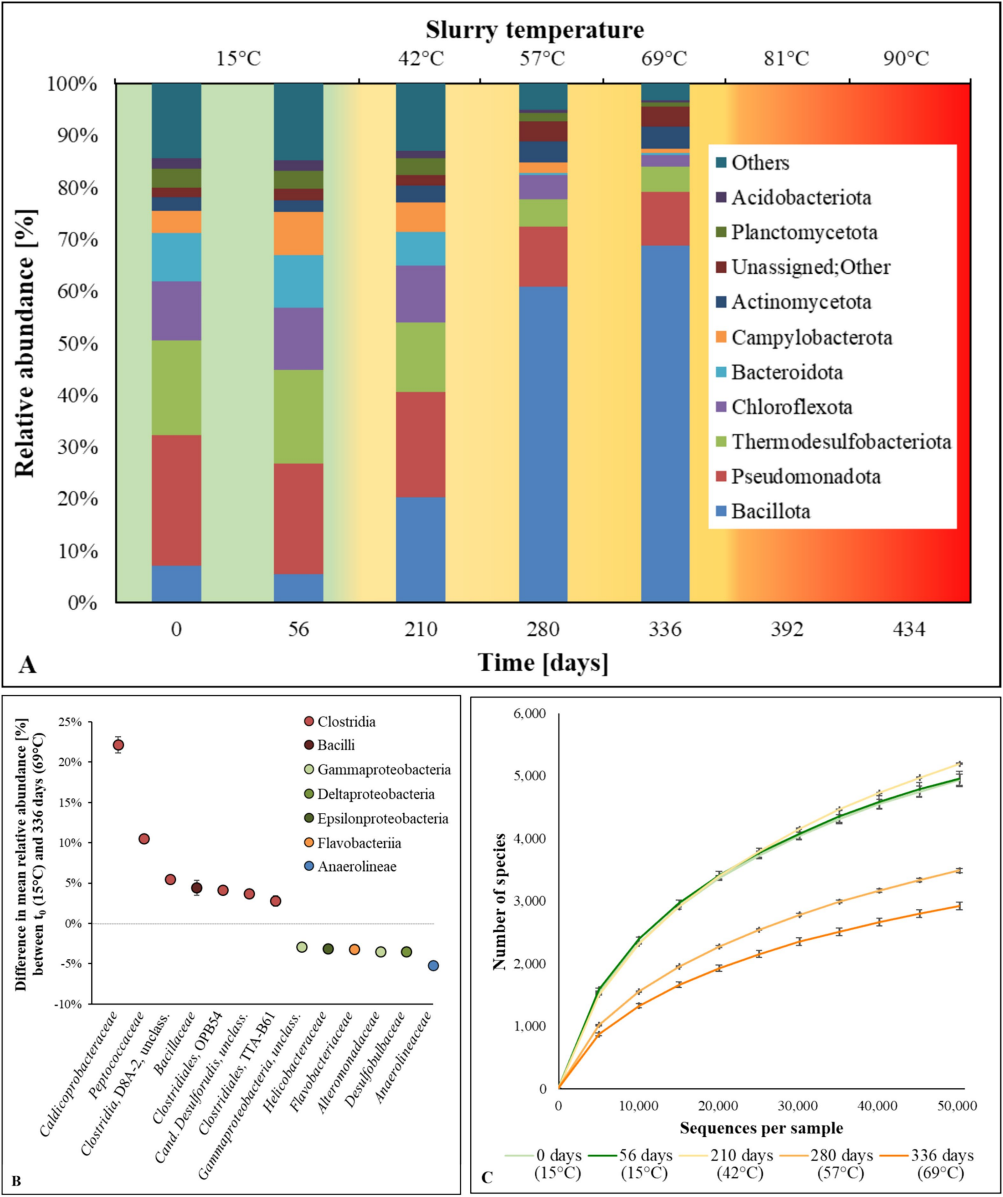
Changes in prokaryotic community composition during sequential heating. **(A)** Relative abundance data at the phylum level (Slurry I). Colored background indicates meso- (green), thermo- (yellow), and hyperthermophilic (red) temperatures range. **(B)** Differences in mean relative abundance of taxa at the family level that cumulatively contribute to more than 50% of the dissimilarity between t_0_ and 336 days (69 °C; SIMPER analysis) are grouped by classes given in the key. **(C)** Alpha diversity rarefaction curves for different time points.

There were only minor changes of the prokaryotic community at the phylum level during the initial 56-day incubation period at 15 °C (Figure 4A). However, during subsequent heating of the slurry, the phylum Bacillota increased from 5.5% relative abundance after 56 days to 68.1% after 336 days (69 °C). This trend was consistent in both slurries (Supplementary Figure 2) and was mainly attributed to increases in the relative abundance of six families affiliated to Clostridia predominantly the Caldicoprobacteraceae (22.1%) and the Peptococcaceae (10.5%), and by one family affiliated to the Bacilli, the Bacillaceae (4.4%; Figure 4B, Supplementary Figure 3A). In contrast, the taxonomic groups showing the strongest decline in relative abundance were the Chloroflexota (Anaerolineaceae 5.2%), Desulfobacterota (Desulfobulbaceae, 3.5%), and Pseudomonadota (Alteromonadaceae, 3.5%). Both Thaumarchaeota and Euryarchaeota were detected throughout sequential heating (1.8 and 1.4% at t_0_, respectively) decreasing in relative abundance to 0.8 and 1.0%, respectively. This moderate decrease can be attributed to the relatively stable abundance of Marine Benthic Group B (on average 0.6%, Supplementary Figure 3B) affiliated to the Thaumarchaeota and an increase in relative abundance during sequential heating of a number of families of the Euryarchaeota (Supplementary Figure 3B) including the methanogenic groups Methanobacteriaceae (from 0.05 to 0.14%), Methanomicrobiaceae (from 0.002 to 0.01%), and the Marine Hydrothermal Vent Group (0.20 to 0.25%). Bacterial sulfate reducers of the Desulfobacterota were detected throughout the experiment decreasing from 13.9% at 0 days to 4.0% after 336 days (69 °C) with the most prominent families being the Desulfobulbaceae and Desulfobacteraceae (Supplementary Figure 3C), the latter still representing 2.0% of the total prokaryotic community at thermophilic temperatures (57 °C to 69 °C).

Some changes in relative abundance occurred during the initial 56-day incubation period. In total, 172 families increased while 223 decreased. The largest increase in relative abundance by two orders of magnitude was found for the Campylobacteraceae (0.004% at 0 days to 0.75% at 56 days, Supplementary Figure 3D), due to an increased number of OTUs affiliated to the genera *Arcobacter* (0.6% at 56 days) and *Sulfurospirillum* (0.19% at 56 days). The Helicobacteraceae doubled their relative abundance but represented a larger proportion of the total community during the initial incubation period increasing from 4.1 to 7.4% after 56 days (Supplementary Figure 3D). Another family that increased in relative abundance was the Hyphomonadaceae (from 0.0013 to 0.032% after 56 days) largely caused by members of the genus *Hyphomonas*. OTUs affiliated to the archaeal ammonia oxidiser *Nitrosopumilus* also increased (from 0.0007 to 0.0033%).

Principal component analysis showed a clear separation of the microbial communities present at temperatures 15 °C and 42 °C compared with the communities present at 57 °C and 69 °C (PERMANOVA *p* = 0.001; Supplementary Figure 4). Hence, the communities at the lowest thermophilic temperature (42°) were more similar to the mesophilic community than to the community at the higher thermophilic temperature range. For slurry I, the similarity between microbial communities at ≤42 °C was 81.9% compared with 75.3% for communities present at ≥57 °C. The two groups only shared 37.2% similarity between them. Additionally, principal component analysis revealed that the communities in the two parallel incubated slurries were similar to each other (68.5% similarity at day 0 and 73.2% similarity at day 336) and thus would likely have undergone similar microbial succession (Supplementary Figure 4).

Unfortunately, community composition profiles for the highest incubation temperatures (81 °C and 90 °C) were not obtained. This was likely caused by a combination of low DNA concentrations (0.28 ng μL^−1^ for 392 days and 0.12 ng μL^−1^ for 434 days) and the likely formation of PCR inhibitors (Webster et al., 2006) due to high slurry temperatures (Roussel et al., 2011; Brandt and Brandt and House, 2016). Alpha diversity remained relatively stable for the first 56 days (15 °C) and increased slightly after 210 days (42 °C) before decreasing with increasing temperature (Figure 4C, Supplementary Table 2). The number of OTUs detected in the slurries at day 336 (69 °C) was 2,917 representing only 59.2% of OTUs present at t_0_ (4,927 OTUs).

### 3.4 Potential metabolic activity after sequential heating to 90 °C

**Figure 5:**
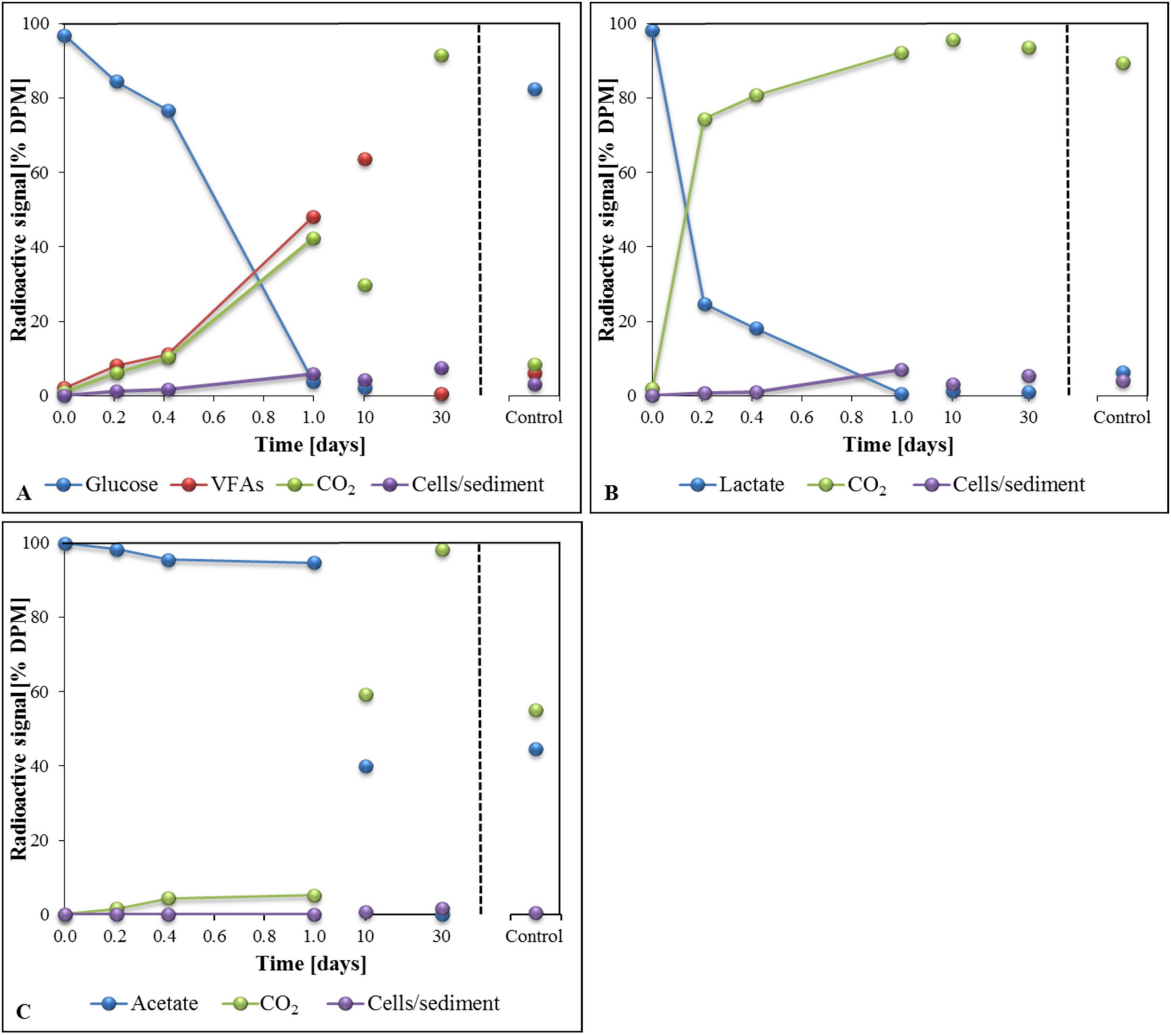
Potential metabolic activity after sequential heating to 90 °C. Metabolic removal of added radiolabelled substrates and production of volatile fatty acids (VFAs, [acetate, lactate, formate]), gaseous CO_2_, and uptake into cells or binding to sediment particles for **(A)** glucose (2.2 nM), **(B)** lactate (3.8 nM), **(C)** acetate (6.7 nM).

In order to confirm that cells were metabolically active at the highest incubation temperature (i.e., 90 °C), ^14^C-labelled substrates (glucose, acetate, and lactate, 2.2 to 6.7 pmol μL^−1^) were added to slurry subsamples. These organic substrates were metabolised rapidly by the microbial community confirming its viability and activity (Figure 5). Glucose was degraded at a rate of 2.0 pmol μL^−1^ d^−1^ (Table 2) and volatile fatty acids (i.e., acetate, lactate, and formate) and carbon dioxide were produced. Subsequently, the produced VFAs were completely consumed after 30 days of incubation and 91.4% of the radioactive signal was detected as carbon dioxide while 7.6% was detected in the particulate sediment fraction of the slurry indicating cellular incorporation or adhesion to the sediment particles (Figure 5A). The two-step degradation of glucose to VFAs and carbon dioxide, and the subsequent degradation of VFAs to carbon dioxide indicates that a syntrophic relationship between glucose-fermenting and acetate consuming members of the community existed.

Added lactate was degraded rapidly at a rate of 3.3 pmol μL^−1^ d^−1^ and only carbon dioxide was detected as a product (Figure 5B). No acetate was detected at any of the time points analysed and cellular incorporation or sediment adhesion was lower than for glucose after 30 days (5.5%). Acetate added was degraded after a lag phase of more than 1 day and was completely consumed between 10 and 30 days (Figure 5C). Only 1.8% of the signal was detected in the filtered cells and sediment and the remaining isotope was present as carbon dioxide. Using the available data, the rate of acetate removal was estimated as 0.7 pmol μL^−1^ d^−1^, although this is likely an underestimation. The metabolic turnover of acetate here also occurred over a similar timeframe in the original slurry (Figure 2B).

Interestingly, concentrations of added radiolabelled substrates decreased to some extent in vials that were autoclaved during the 30 days of incubation. Glucose and acetate concentrations decreased by 17.6 and 55.5%, respectively (Figures 5A,C) whereas lactate was almost completely removed after 30 days (93.6%; Figure 5B). This suggests that autoclaving did not completely inactivate all high temperature-adapted microorganisms or spores.

## 4 Discussion

In order to investigate the physiological responses of an estuarine near-surface sediment microbial community to increasing temperature, this study comprehensively analysed the viability, culturability, diversity, and potential metabolic activity of a surface microbial community exposed to a progressive temperature increase.

**Table 2:** Potential metabolic rates based on cell counts.

Rate based on cell counts [pmol day^−1^]	Glucose amended		Acetate amended	Lactate amended
	Glucose	VFA		
Volume [mL]	2.0	0.3	0.7	3.3
Total cell count	1.0 × 10^−6^	1.7 × 10^−7^	3.5 × 10^−7^	1.7 × 10^−6^
FISH count	3.9 × 10^−6^	6.3 × 10^−7^	1.3 × 10^−6^	6.4 × 10^−6^
MPN	8.1 × 10^−3^	6.8 × 10^−4^	1.4 × 10^−3^	7.1 × 10^−3^

FISH count = bacteria only (EUB338-I probe); Rates calculated from MPNs are based on different metabolic groups. Rates for glucose degradation are based on MPN values obtained from heterotrophs (YPG medium); rates for VFA in the glucose amended slurry and acetate amended slurry are based on SRP and methanogen medium; rates for lactate degradation are based on SRP medium.

### 4.1 Response of an intertidal sediment microbial community to sequential heating

The detection of microorganisms in relatively high cell numbers throughout sequential heating of mesophilic sediments suggests resilience of the microbial communities towards elevated temperatures. Surprisingly, MPN counts obtained at a specific temperature remained high even if the slurries were then heated to a higher temperature. For example, MPN counts after incubation at 40 °C remained high in subsamples incubated at (hyper-) thermophilic temperatures (Supplementary Figure 1). This survival of some subpopulations (i.e., mesophiles) is most likely due to spore formation or the presence of microorganisms with an extreme growth temperature range (Bouanane-Darenfed et al., 2013). This suggests a possible mechanism for inoculation of the deep biosphere by the latent thermophilic subpopulation present within the near-surface sediment microbial community, which is likely to comprise of endospores from thermophiles that are frequently detected in cold sediments (Isaksen et al., 1994; Hubert et al., 2010; Bell et al., 2018).

FISH detectable bacterial cell numbers in the slurries were indicative of viability, and comparable to those reported for marine sediments (Biddle et al., 2006a; Buongiorno et al., 2017), but much lower than those in near-surface sediments of intertidal mud flats (0–0.05 mbsf, Llobet-Brossa et al., 1998; ≤0.15 mbsf, Pernthaler et al., 2002). The number of Bacteria detected by FISH were consistently two orders of magnitude below the total cell count except in the range of hyperthermophilic temperatures (Figure 2). Given the FISH probe used here does not cover all bacterial taxa (Daims et al., 1999), omission of the phyla Planctomycetota and Verrucomicrobiota could have contributed to this phenomenon. However, these taxa constituted only small proportions to the overall diversity in the heated slurries (Figure 4). Furthermore, at these high temperatures the majority of cells may have contained fewer ribosomes than are required for the detection by FISH (Hoshino et al., 2008), suggesting that a proportion of the microbial community was less active than quantified by this method. Consequently, the decrease in viability reported here may represent a steeper decline than would have been observed using the more sensitive CARD-FISH technique (Pernthaler et al., 2002).

The different degradation rates of added carbon substrates in combination with the culturability data (Table 2; Figure 3) indicated that different metabolic clades of microorganisms were active at hyperthermophilic temperatures. Added substrates were fully metabolised to CO_2_ with only little carbon being retained in the cells or bound to sediment (maximum 7.6% after 30 days in the glucose-amended slurry; Figure 5). This is consistent with observations from cells inhabiting oligotrophic environments where the mean cell carbon content is low (Lever et al., 2015) suggesting that bioavailable organic carbon is used for energy conservation rather than biomass synthesis or storage. Interestingly, metabolic activity was also detected in autoclaved control vials. This was most pronounced for added radiolabelled lactate (Figure 5B), suggesting that perhaps ultra-resistant spores of hyperthermophilic sulfate reducers may have survived autoclaving as previously reported (O'Sullivan et al., 2015). Although the autoclaved vials may not represent a truly abiotic control, any taxa that survived as spores likely originated from the 90 °C incubation period up to day 434 of the experiment. Consequently, their activity may have been effectively observed twice, first as part of the active hyperthermophilic community in the unautoclaved vials and again following their germination in the autoclaved vials.

### 4.2 Responses of community composition to increasing temperatures and dominance of thermophilic Bacillota at high temperatures

The diversity of the marine deep biosphere is relatively high (Ruff et al., 2024) and differs between sites (Fry et al., 2008) despite the similar and predictable biogeochemical zonation of marine sediments (Froelich et al., 1979). Consequently, other factors such as carbon availability (amount and type), burial rate, and temperature must also govern the microbial diversity, allowing both phylogenetic and metabolic diversity to be maintained (Roussel et al., 2015). During this experiment, prokaryotic diversity initially increased up to 210 days (42 °C; Figure 4C; Supplementary Table 2) accompanied by an increase in total cell counts (Figure 2) likely caused by the homogenisation of previously stratified sediments and a concomitant redistribution of both electron donors and acceptors, and the slow transition to thermophilic temperatures which may have activated some of the more recalcitrant carbon compounds (Parkes et al., 2007). Continued heating eventuated in the overall dominance of thermophilic members of the bacterial classes Clostridia and Bacilli, specifically members of the Caldicoprobacteraceae, Peptococcaceae, and Bacillaceae (Figures 4A,B) most of which are able to form spores. Spores of thermophilic microorganisms have been detected in cold marine sediments (Hubert et al., 2009; Volpi et al., 2017; Gittins et al., 2022) an even been shown to germinate and become metabolically active when conditions become suitable (Hubert et al., 2010; O'Sullivan et al., 2015). This seemingly occurred here. However, the extent to which members of the Bacillota dominated the prokaryotic community composition in sediment slurries at temperatures above 57 °C is somewhat unusual and a similar dominance has only been reported for coal-bearing marine sediments at depth (Inagaki et al., 2015; Petro et al., 2017). Their presence may also be attributed to extracellular DNA from dead bacteria.

The Caldicoprobacteraceae were the most prominent taxa at 69 °C. To date, this family only contains one validly described genus (*Caldicoprobacter*) and the type species isolated from sheep faeces has been reported to form spores (Yokoyama et al., 2010). In contrast, no spore formation was observed for isolates obtained from hot springs (Bouanane-Darenfed et al., 2011; Bouanane-Darenfed et al., 2013) and thermophilic anaerobic digesters (Azizi et al., 2016; Poirier et al., 2016). *Caldicoprobacter* spp. are strict anaerobes that ferment a range of carbon compounds and thus do not require any inorganic electron acceptors. The main glucose fermentation products are acetate, lactate and hydrogen. Concentrations of acetate and hydrogen increased with increasing slurry temperature (Figures 1A,B) whereas lactate was not detected at temperatures above 48 °C. Added lactate was rapidly removed in radiotracer experiments at 90 °C (less than 1 day, Figure 5B) suggesting syntrophic relationships between the fermenting Caldicoprobacteraceae and lactate-utilising microorganisms.

The Peptococcaceae contain several genera comprising thermophilic species, such as *Desulfotomaculum* which is commonly detected in marine sediments (Vandieken et al., 2006; Fichtel et al., 2012; O'Sullivan et al., 2015). *Desulfotomaculum* spp. are sulfate reducers that can use a variety of electron donors, including a few species able to use acetate (Pikuta et al., 2000; Widdel, 2006). Despite constituting 5.4% to the overall prokaryotic community and with both acetate and sulfate being present at 69 °C (Figure 1C), the metabolic activity of *Desulfotomaculum* spp. appears to have been low as no increased removal of acetate or sulfate occurred concomitant to the increase in relative abundance of the Peptococcaceae. In radiotracer experiments conducted here at 90 °C, acetate removal was slow (between 10 to 30 days; Figures 5A,C) in both the acetate-amended and glucose-amended subsamples as well as in the original slurry (Figure 1C).

*Desulforudis* represented 3.6% of the total prokaryotic community at 69 °C. This taxon was highly abundant in hot (65 °C) basaltic crustal fluids of the of the Juan de Fuca Ridge (Jungbluth et al., 2012) and isolated from an aquifer in Siberia (Karnachuk et al., 2019). This taxon is capable of sulfate reduction and nitrogen fixation while utilising both organic and inorganic carbon sources (Karnachuk et al., 2019). It has also been speculated that heterotrophic carbon sources may include dead cells (Chivian et al., 2008) which would have been abundant in this experiment given the decline in total cell numbers above 42 °C (Figure 2, Table 1). Necromass amino acid carbon is one of the less refractory carbon sources in the marine deep biosphere and thus preferentially degraded over bulk organic matter (Lomstein et al., 2012; Braun et al., 2017). It follows that other organic carbon compounds including sugars, fatty and nucleic acids released by dying cells should be equally available for uptake by any viable chemoorganotroph unless absorbed by sediment particles and incorporated into the kerogen pool. In this experiment, necromass would have been expected to be produced whenever temperatures were increased above the maximum limit for specific groups of microorganisms (e.g., mesophiles). Indeed, total cell counts started to decrease in the thermophilic temperature range (>42 °C; Figure 2) and this accelerated in the hyperthermophilic temperature range (>70 °C, Figure 2). The variety of compounds released by cell death has the potential to stimulate a range of different taxa depending on their carbon source preference. For example, the aforementioned Caldicoprobacteraceae are known to utilise sugars but not proteinaceous compounds (Bouanane-Darenfed et al., 2014). The latter could be degraded by extracellular proteases commonly produced by Bacillaceae (e.g., *Bacillus* spp., Priest, 1977; *Geobacillus* spp., Wissuwa et al., 2016) while *Syntrophomonas* spp. (Supplementary Figure 3A) are capable of degrading short and long-chain fatty acids producing hydrogen and acetate (Roy et al., 1986; Zhang et al., 2005) which may have also contributed to the increasing acetate concentrations in the slurry (Figure 1C). Our findings support previous results that increasing temperature provides carbon sources (e.g., acetate) from organic matter (Wellsbury et al., 1997) and necromass (Lomstein et al., 2012) which in turn allows the persistence of a diverse prokaryotic community with syntrophic interactions. The combination of biogeochemical and sequencing data highlights syntrophic interactions that may occur in marine sediments at depth and account for the majority of carbon turnover.

### 4.3 Activity of sulfate reducers and methanogens

Several studies reported more rapid sulfate removal in sediment slurries at mesophilic temperatures (Kevorkian et al., 2018) and when subjected to heating to 50 °C – 60 °C (Parkes et al., 2007; Parkes et al., 2011) than occurred during this experiment. Here, sulfate consumption was continuous throughout sequential heating but with several rapid concentration increases (Figures 1C,D), suggesting replenishment of the sulfate pool either by anaerobic oxidation of pyrite with iron oxide minerals (Bottrell et al., 2000) or bacterial sulfur oxidation (Inagaki et al., 2004). Nevertheless, the sulfate reduction rates in the two slurries (0.022 mmol L^−1^ d^−1^) are comparable with rates reported from other sediments (Fossing and Jørgensen, 1990; Ferdelman et al., 1999; O'Sullivan et al., 2013). This continued activity is consistent with MPN estimates of culturable SRPs throughout sequential heating (Figure 3) and supported by the high abundance of sulfate reducers at mesophilic temperatures (Supplementary Figure 3C) with a shift to an increasing abundance of potentially sulfate-reducing *Desulfotomaculum* spp. within the family Peptococcaceae (Supplementary Figure 3A) at thermophilic temperatures. The lack of a typical sulfate reduction time course as described for other sediment slurries and marine sediments could be attributed to the limited supply of easily degradable carbon substrates and thus electrons for sulfate reduction at low temperatures.

Methane accumulated with temperature increase as previously observed in other studies (e.g., Conrad, 2023). However, this sharp increase in methane was limited to the first 42 to 56 days of the experiment (i.e., up to 15 °C; Figure 1A). The initial increase in methane concentrations coincided with an increase in culturable methanogens (Figure 3) but only a slight increase in the relative abundance of methanogenic 16S rRNA genes (e.g., Methanosarcinaceae; Figure 4A and Supplementary Figure 3B). Roussel et al. (2015) reported that at these low temperatures methane can potentially be produced by three different processes using bicarbonate and hydrogen or C_1_ compounds as substrates with rates in the picomolar and nanomolar range per day and cubic centimetre of sediment, respectively. In addition, acetoclastic methanogenesis may also occur (picomolar range) but acetate is likely to be used for putative metal reduction (nanomolar range). Unlike heating experiments with coastal sediments from Portishead, UK (Roussel et al., 2015) where slurries were incubated at the same temperature for extended periods of time (up to 100 days) to allow establishment of predominant metabolisms, here temperature was sequentially increased every 2 weeks. This time length may have been insufficient for slow-growing methanogens to adapt to the changing conditions, as the ‘windows of opportunity’ for the respective metabolisms may have been too short. The initial methane production occurred at 15 °C (Figure 1A), similar to the sediment temperature at the time of collection (12 °C), and as such a mesophilic methanogenic community would have been well established *in situ*. Increasing the temperature by 3 °C every 2 weeks may have been too rapid to allow for different methanogenic processes to be manifested as was shown by Roussel et al. (2015). The decrease in methane concentrations observed from day 56 onwards could be attributed to anaerobic oxidation of methane coupled to sulfate reduction (Boetius et al., 2000). Although sulfate-reducing taxa typically associated with this process (i.e., Desulfobacteriaceae and Desulfobulbaceae) were present during this period of decline (Supplementary Figure 3C), members of the ANME-1 and ANME-2 archaea were detected only at extremely low abundances (<0.01% between 56 and 210 days, across 15–42 °C), suggesting that this process may have been limited or occurring at low levels under the conditions studied.

## 5 Limitations of this study

This study has some limitations which may impact its ecological conclusions. Firstly, only two replicate slurries were investigated. Despite this limited replication, the two replicates showed good agreement in both their biogeochemical profiles (Figure 1) and microbial community development (Supplementary Figures 2, 4), which gives credibility to the reproducibility of this study. Secondly, the FISH probe EUB338-I does not target all bacterial taxa. However, this limitation was mitigated through complementary community profiling. Lastly, the experimental approach of using increasing temperature as a proxy for processes that occur during burial over geological timescale remains an area of contention. In this study, the approach used did not reproduce microbial communities comprising of well-characterised deep biosphere taxa, such as Chloroflexota and Atribacterota (Parkes et al., 2014). Instead, it selected for a cryptic thermophilic ‘seed bank’, represented by members of the bacterial class Clostridia which were present within the original mesophilic near-surface sedimentary community.

## 6 Conclusion

Our results highlight successive changes of surface sediment microbial communities in response to increasing temperatures during which some of its members maintain cellular activity and viability up to at least 90 °C. Without allochthonous substrates, sulfate reduction and methane production were low. Nevertheless, fermentation of sediment organic matter and necromass produced by increased heat stress may have continuously supplied low concentrations of electron donors supporting microbial metabolism and energy production in heterotrophic taxa at high temperatures. These findings are consistent with the presence of large cell populations in high-temperature sub-seafloor sediments and help understand how the marine deep biosphere is sustained.

## Data Availability

The datasets presented in this study can be found in online repositories. The names of the repository/repositories and accession number(s) can be found in the article/Supplementary material.

## Footnotes

1
https://www.ebi.ac.uk/ena

2
https://real-statistics.com

## In memoriam

This paper is dedicated to Prof. John R. Parkes and Dr. Barry Cragg, both were pioneers in the field of deep biosphere microbiology, and their influence on this paper was as great as many of their other studies. They are sadly missed.
