# Supplementary material for: Surface sediment microbial communities remain viable, culturable, and metabolically active during sequential heating
Source: Front Microbiol. 2026 Jun 10;17:1840877. doi: 10.3389/fmicb.2026.1840877 (PMC13291160; doi:10.3389/fmicb.2026.1840877)
Supplement: Supplementary file 1 [file Data_Sheet_1.pdf]

SUPPLEMENTARY INFORMATION

Surface sediment microbial communities remain viable, culturable, and metabolically active during sequential heating.

Falko Mathes[a,b], Erwan G. Roussel[a,c], Barry A. Cragg[a,†], Andrew J. Weightman[d], Henrik Sass[a*], R. John Parkes[a,†], Gordon Webster[d,*]

[a]School of Earth and Environmental Sciences, Cardiff University, Park Place, Cardiff, CF10 3AT, Wales, UK

[b]UWA School of Agriculture and Environment, The University of Western Australia, 35 Stirling Highway, Perth, WA 6009, Western Australia, Australia

[c]Univ Brest, Ifremer, Biologie et Ecologie des Ecosystèmes marins Profonds (BEEP), F-29280 Plouzané, France

[d]Microbiomes, Microbes and Informatics (MMI) Group, School of Biosciences, Cardiff University, Museum Avenue, Cardiff, CF10 3AX, Wales, UK

[†]Deceased

*** Correspondence:**

Gordon Webster: WebsterG@cardiff.ac.uk

Henrik Sass: SassH@cardiff.ac.uk

Supplementary Table 1: Cultivation efficiencies at *in situ* slurry temperature based on total cell counts.

Time [days]	Temperature [°C]	Total cell count [cells mL^{-1}]	Heterotrophs [%]	SRPs [%]	Methanogens [%]
0	15	1.2×10^{8}	0.12	0.009	3.1×10^{-6}
56	15	1.6×10^{8}	0.68	0.15	0.0046
210	42	2.5×10^{8}	0.04	0.06	1.8×10^{-4}
280	57	1.7×10^{8}	1.37*	0.26*	0.014*
336	69	1.2×10^{8}	0.0009*	0.0020*	0.002*
420	87	3.5×10^{6}	0.0025*	0.0025*	5.4×10^{-4}*
434	90	1.9×10^{6}	0.01	0.025	4.2×10^{-4}

* Indicates that data was taken from MPN incubated at the previous slurry temperature due to oxidation of the MPN incubated at the respective *in situ* slurry temperature.

Supplementary Table 2: 16S rRNA gene sequencing metrics and diversity indices.

Time	Temperature	Number of	Number of	Alpha diversity indices			
[days]	[°C]	sequence reads	OTUs	Observed species	Chao1	Shannon	PD
0	15	77,820 (±2,038)	8,617 (±607)	4,927 (±100)	6,153.8 (±234.5)	9.62 (±0.05)	244.5 (±0.9)
56	15	76,864 (±1,208)	8,569 (±424)	4,959 (±116)	6,418.3 (±392.5)	9.69 (±0.05)	246.9 (±3.5)
210	42	79,803 (±702)	9,481 (±96)	5,191 (±10)	6,369.8 (±152.0)	9.47 (±0.01)	237.1 (±1.0)
280	57	69,423 (±2,957)	6,392 (±508)	3,486 (±34)	4,759.8 (±58.5)	8.05 (±0.03)	170.2 (±2.7)
336	69	68,022 (±1,306)	5,597 (±30)	2,917 (±64)	4,007.8 (±97.6)	7.56 (±0.10)	148.0 (±2.2)
392	81	n.d.	n.d.	n.d.	n.d.	n.d.	n.d.
434	90	n.d.	n.d.	n.d.	n.d.	n.d.	n.d.

Standard errors (in parenthesis), Alpha diversity measures were assessed after rarefaction to 50,300 sequences per sample. n.d. = no data.

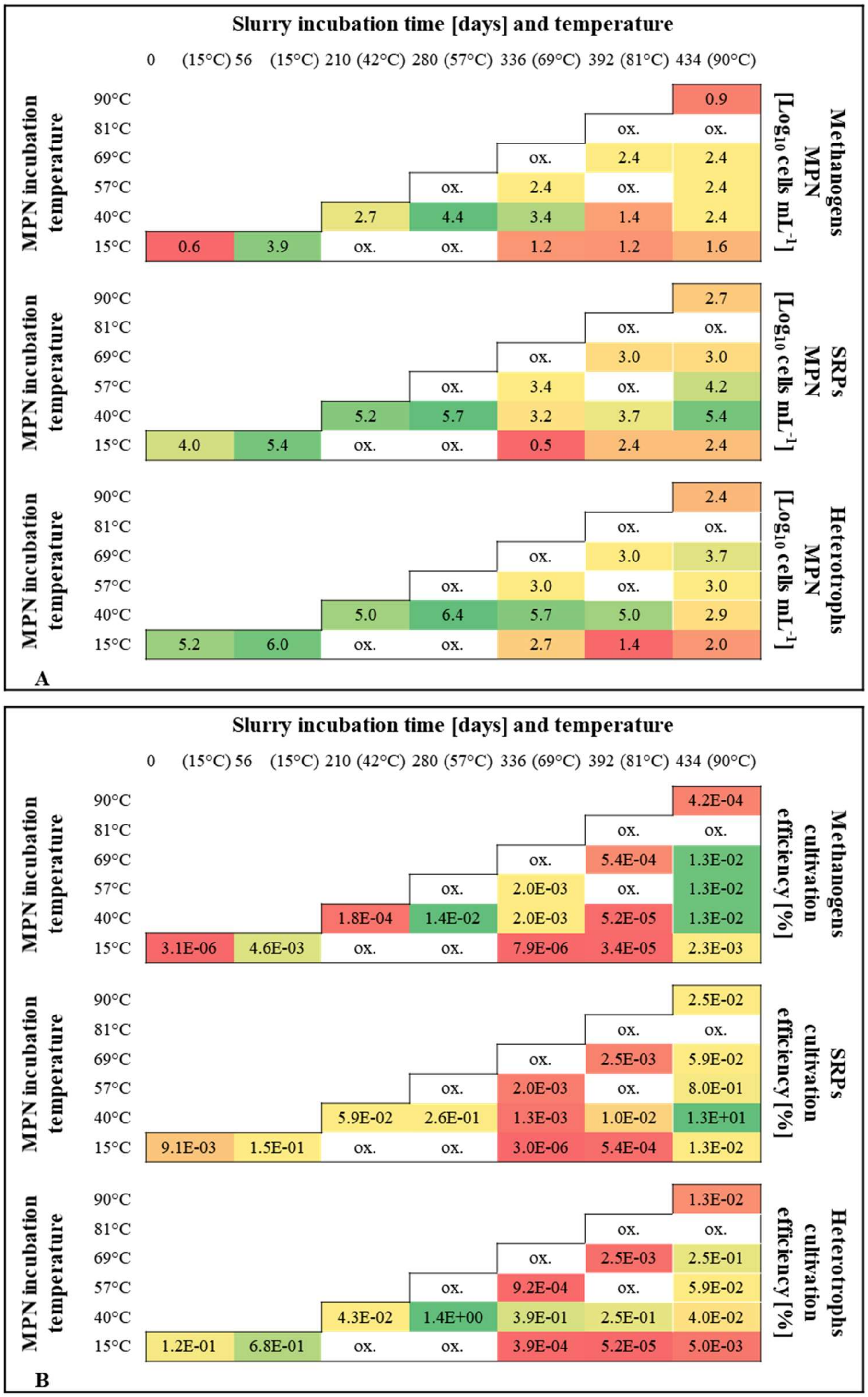

Supplementary Fig. 1. Culturability matrix for different metabolic groups during sequential heating incubated at different temperatures. (**A**) Log MPN values and (**B**) cultivation efficiencies (based on AODC total counts) for different metabolic groups (heterotrophs, sulfate reducers, and methanogens, right hand side) are given for different sampling points throughout the sequential heating experiment (top) incubated at different temperatures (left hand side). Confidence limits (95%) were determined but have been omitted for clarity. n.d. = no data due to oxidation of the MPN 96 multi-well plate during incubation.

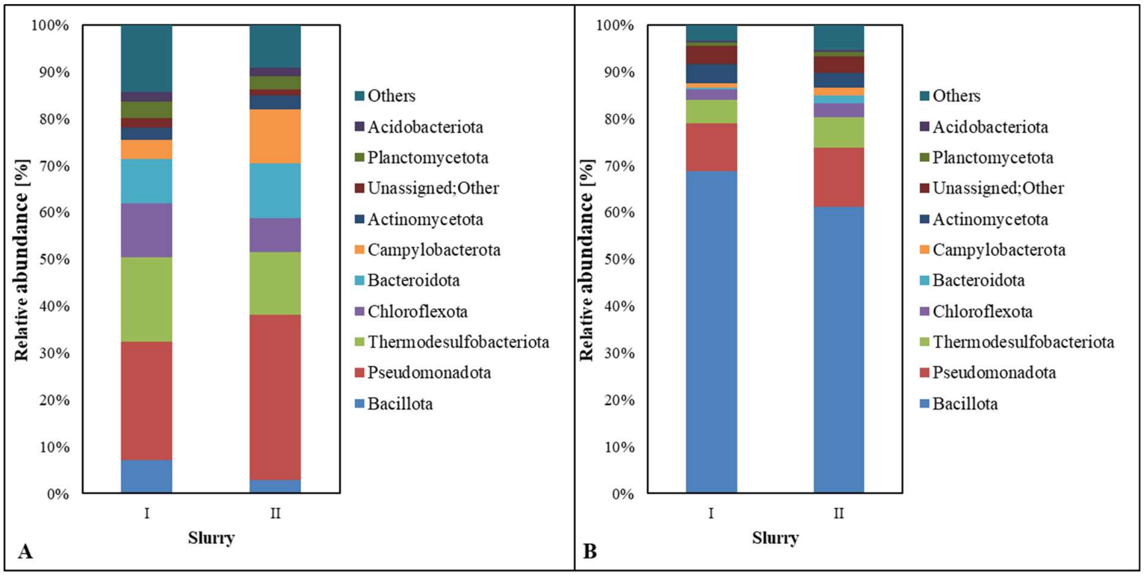

Supplementary Fig. 2. Prokaryotic community composition at the phylum level for two parallel slurries. Despite some differences in the relative abundance profiles of the two parallel incubated slurries at the phylum level at t_0 (15°C, 68.3% shared similarity) **(A)** and at 366 days (69°C, 65.6% shared similarity) **(B)**, the trend between the two time points was consistent. Sequential heating resulted in an increased relative abundance of *Bacillota*.

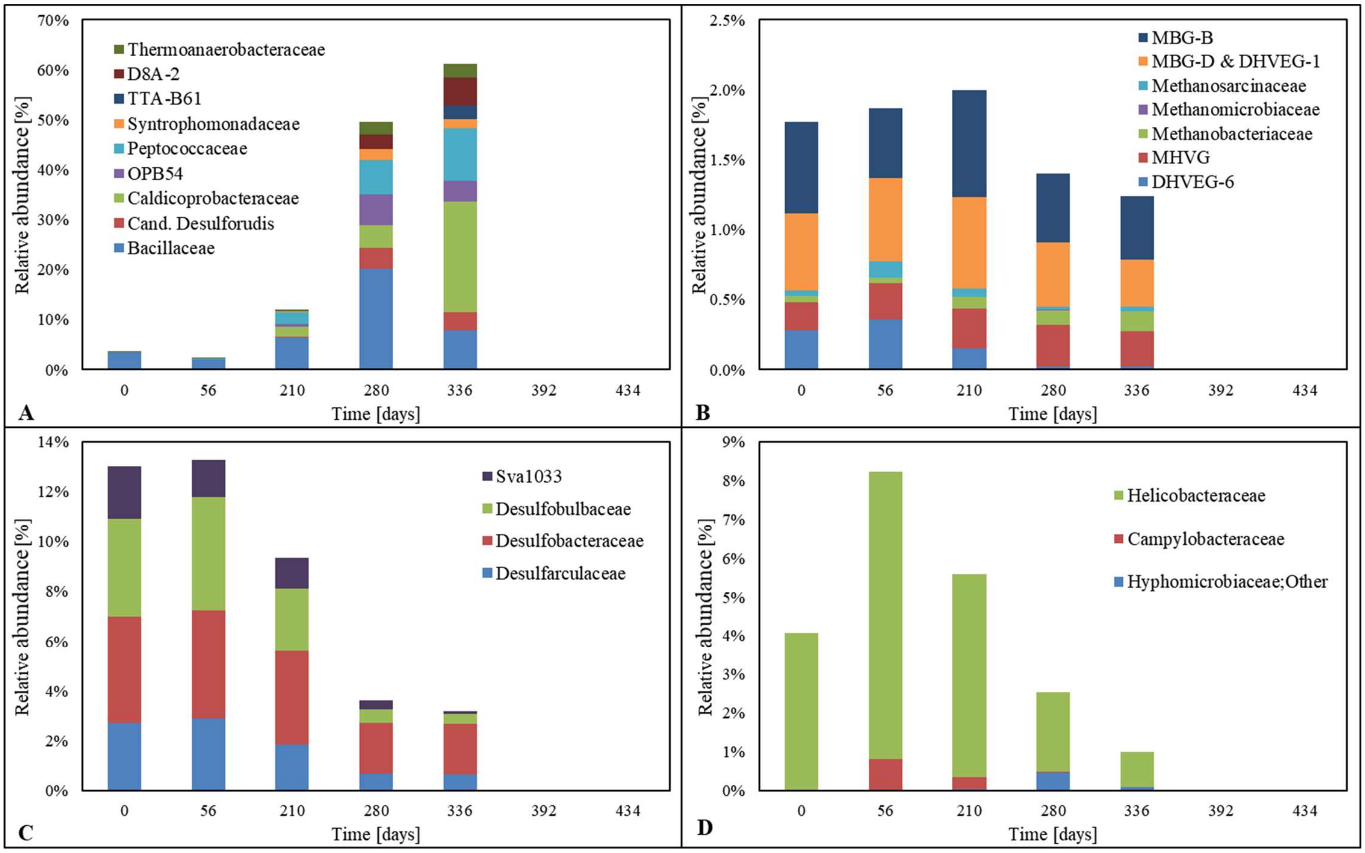

Supplementary Fig. 3. Prokaryotic community composition at the family level for selected taxa. The community composition responded dynamically to increasing temperatures with different taxa showing different relative abundances at certain temperatures. **(A)** *Bacilli* and *Clostridia* (*Bacillota*). **(B)** *Methanobacteriota* and *Nitrososphaerota*. **(C)** *Desulfarculales*, *Desulfobacterales* and *Desulfuromonadales* (*Desulfobacterota*). **(D)** *Campylobacterota* and *Pseudomonadota*.

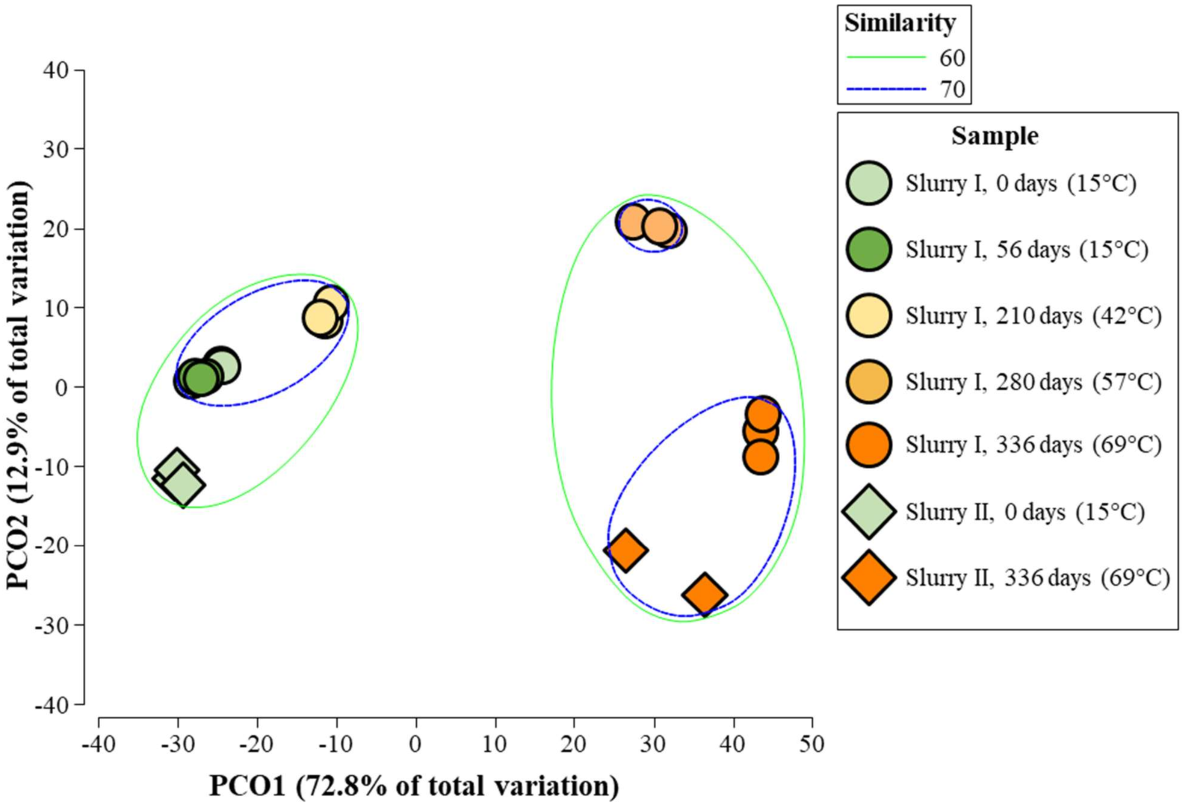

Supplementary Fig. 4. Beta diversity showing changes in microbial community structure during sequential heating of the slurries. Principal component analysis (PCoA) shows a clear distinction between samples collected from temperatures ≤42°C and ≥57°C. Additionally, the PCoA highlights the similarity of samples collected from the two slurries at the same timepoints (i.e. days 0 and 336).
